# Temporal trend and spatial clustering of the dengue fever prevalence in West Java, Indonesia

**DOI:** 10.1016/j.heliyon.2022.e10350

**Published:** 2022-08-25

**Authors:** Ilham Saiful Fauzi, Nuning Nuraini, Regina Wahyudyah Sonata Ayu, Bony Wiem Lestari

**Affiliations:** a Department of Accounting, Politeknik Negeri Malang, Malang, Indonesia; b Department of Mathematics, Faculty of Mathematics and Natural Sciences, Institut Teknologi Bandung, Bandung, Indonesia; c Center for Mathematical Modeling and Simulation, Institut Teknologi Bandung, Bandung, Indonesia; d Department of Mathematics, Faculty of Mathematics and Natural Sciences, Universitas Palangka Raya, Palangkaraya, Indonesia; e Department of Public Health, Faculty of Medicine, Universitas Padjadjaran, Bandung, Indonesia; f Department of Internal Medicine, Radboud Institute for Health Sciences, Radboud University Medical Centre, Nijmegen, the Netherlands

**Keywords:** Dengue fever, Infectious disease modeling, Temporal trend, Richards model, Spatial clustering, Hot spot analysis

## Abstract

Dengue fever is a notable vector-borne viral disease, currently becoming the most dreaded worldwide health problem in terms of the number of people affected. A data set of confirmed dengue incidences collected in the province of West Java has allowed us to explore dengue's temporal trends and spatial distributions to obtain more obvious insights into its spatial-temporal evolution. We utilized the Richards model to estimate the growth rate and detect the peak (or turning point) of the dengue infection wave by identifying the temporal progression at each location. Using spatial analysis of geo-referenced data from a local perspective, we investigated the changes in the spatial clusters of dengue cases and detected hot spots and cold spots in each weekly cycle. We found that the trend of confirmed dengue incidences significantly increases from January to March. More than two-third (70.4%) of the regions in West Java had their dengue infection turning point ranging from the first week of January to the second week of March. This trend clearly coincides with the peak of precipitation level during the rainy season. Further, the spatial analysis identified the hot spots distributed across central, northern, northeastern, and southeastern regions in West Java. The densely populated areas were likewise seen to be associated with the high-risk areas of dengue exposure. Recognizing the peak of epidemic and geographical distribution of dengue cases might provide important insights that may help local authorities optimize their dengue prevention and intervention programs.

## 1 Introduction

Dengue fever is regarded as the most rapidly spreading disease and has become a significant health issue in tropical and sub-tropical countries [1], [2], [3]. It is one of the most prevalent arthropod-borne illnesses caused by virus infection with any of the four serotypes [4]. The primary vector of virus transmission is the *Aedes* mosquito, with humans and primates as their natural host. Dengue cases have affected more than 100 countries globally with a 30-fold increase in the past five decades [5], [6]. Tens of millions of dengue cases are reported, and tens of thousands of fatality cases occur annually [7]. It is estimated that about two-thirds of the human population reside in areas infested with dengue vectors [8]. The high prevalence, the limitations of vaccines, and the lack of particular treatment have lead dengue fever to become a public health threat, causing widespread concern [9].

Nowadays, the Asia and Pacific regions are the highest-risk geographical areas for dengue infection in which virus transmission easily expands [10]. Located in this area, Indonesia has over 100,000 reported dengue cases each year and has become one of the riskiest areas of dengue exposure [11]. Tropical climate conditions support mosquito growth so the transmission cycle between hosts and vectors is likely to continue with increasing intensity. The seasonal variation between the rainy and dry seasons is associated with the annual variety of dengue incidences [12]. Breeding site expansion during the rainy season determines the abundance of the vector population. Subsequently, it increases the risk of virus transmission, whereas the lack of egg-laying space during drought periods favors the reduction of mosquito population size [13].

The province of West Java is located in the central-southern region of Indonesia, adjacent to the capital city of Jakarta. With a population of approximately 50 million, in which more than three-fourths are concentrated in urban and semi-urban areas, dengue has become a notable infectious disease in West Java. Transmission in such a large and densely populated area has significant spatial heterogeneity, where infections spread inside as well as towards surrounding locations with less population caused by the intense flow of people [14]. Sustainable dengue transmission requires a minimum population size of approximately 10,000-1,000,000 [15], and dengue epidemics in Southeast Asia are related to cities with populations over 10,000 [16]. The densely populated areas where most economic activities happen are distributed in the central, northwestern, and northeastern regions of West Java. Environmental degradation and rapid urbanization, which might be led by economic development, could have simplified virus importation and vector expansion, which in turn may have encouraged the temporal and spatial distribution of dengue cases.

Some research has been conducted to explain the traveling wave type pattern of dengue incidences around the world. Akter et al. [17] have identified that newly affected areas appear to have been expanding in Queensland over recent years, which signifies potentially increasing risk for unaffected areas in and around hot spot areas, Cairns. Churakov et al. [18] have shown that human mobility is a significant contributor to spatial patterns of dengue in Brazil. In its specific case, the traveling wave starts in the western states, then travels eastward and finally reaches the northeast at the end of a typical dengue season. Finally, the large scale of human suffering caused by virus infection becomes a challenge within the process of identifying risk factors and pinpointing high-risk areas.

At present, since there are no effective treatment options for dengue fever, prevention is the best effort to protect the population from the infection risk. The method to prevent and control dengue virus transmission is limited to suppressing the mosquito population through some preventive measures, including reducing mosquito access to egg-laying sites such as inside and outside water-filled containers as well as using larvicides and adulticides to eradicate mosquitoes. In order to implement preventive actions effectively, the capability to recognize the geographical distribution and heterogeneity of dengue case patterns accurately and detect potential regions in which virus exposure may be responsible for intense infection will provide essential insights that support local health authorities in determining high-risk areas and secure human population. This research aims to evaluate the temporal trend and spatial clustering of dengue incidences in West Java and provide a more obvious description of the spatial-temporal evolution of dengue cases, which could hopefully optimize dengue prevention and intervention management by public health workers and the local government.

## 2 Material and methods

### 2.1 Study site

**Figure 1:**
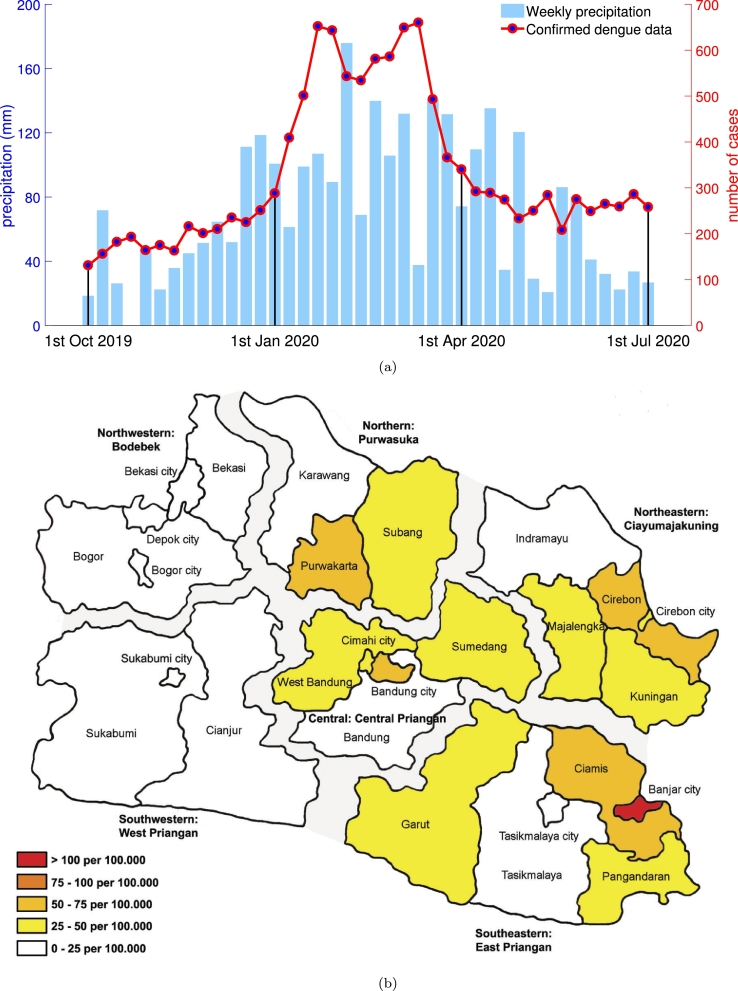
Summary of dengue incidence data recorded by the West Java Health Office: (a) weekly dengue incidence and weekly precipitation, and (b) dengue incidence rate in all districts and cities.

The province of West Java is located at 104°48′E to 108°48′E and 5°50′S to 7°50′S and covers a total area of 35,378 square kilometers. West Java features a tropical climate with a high annual average precipitation of approximately 2,000-4,000 mm. The average temperature expands from 9 °C in the highlands to 34 °C at the coast. West Java is the most populous province in Indonesia, with a total population reaching about 49.94 million people in 2020, or 18.48% of the total population in Indonesia. Currently, 18 districts and 9 cities are under the jurisdiction of West Java. Based on the geographical location, districts and cities in West Java can be classified into six regions as shown in Fig. 1b, i.e., the northwestern region: Bodebek, the northern region: Purwasuka, the northeastern region: Ciayumajakuning, the southeastern region: East Priangan, the central region: Central Priangan, and the southwestern region: West Priangan. The district of Pangandaran has the lowest population density with 398 people per square kilometers, whereas the highest population density is in the city of Cimahi with 15,798 people per square kilometer. A tremendous amount of the population is concentrated in urban and semi-urban areas that are the center of modern economic activities in the central (including Bandung), northwestern (including Bogor, Bekasi, and Depok), and northeastern (including Cirebon) regions. In West Java, the more densely populated areas are more economically progressed and have a more dynamic human population.

### 2.2 Dengue incidence data

Dengue is a disease commonly found throughout the year in West Java, particularly because the environmental condition supports the dengue vector's existence. As part of the dengue management effort, several mosquito control activities such as residual and chemical insecticide spraying are administered by the Dengue Response Team from district/city health offices in the outbreak regions. The number of dengue cases usually starts to increase in October, upon the arrival of the rainy season, and it decreases in May-June, at the end of the rainy season. The raw dengue data used in the present study are the weekly hospitalized dengue indigenous incidences reported in West Java. Since we were unable to obtain the complete dengue data in a year (52 weeks) due to the lack of data availability, we only used a 40-week data period starting from the first week of October 2019 to the first week of July 2020, which affected 13,169 individuals.

Due to the importance of reported dengue cases as the notifiable disease in West Java, dengue data is permanently recorded systematically and continuously by the West Java Health Office (Dinas Kesehatan). Dengue data is anonymized to protect the confidentiality and privacy of the patient. Within 24 hours of diagnosis, the doctors are required to report all lab-confirmed or clinically diagnosed cases. Fig. 1a shows the weekly confirmed dengue cases in West Java, and Fig. 1b shows that high dengue incidence rates are distributed in the central, northern, northeastern, and southeastern regions of West Java.

### 2.3 Mathematical model

To examine temporal trends, the data of weekly reported dengue cases were fitted to the Richards model. In Fig. 1a, the weekly dengue cases in West Java show a single wave and since the dengue epidemic in Indonesia usually starts in the early rainy season (late year) and ends in the early dry season (mid-next year) [19], [20], [21], we assumed that the fitting process would be generating an exactly single wave of cases for all cities and districts in West Java. The form of the Richards model [22], [23] is shown as the following equation:

(1) $$C(t)=K{\left[1+e^{-r\mu \left(t-t_{i}\right)}\right]}^{-1/\mu }$$

C(t) denotes the cumulative number of weekly dengue incidences, with t=0 being the initial week of dengue wave observation. Parameter *K* and *r* are the total numbers of dengue incidences and the cumulative case number's growth rate per capita over this wave, respectively. Further, *μ* signifies the cumulative curve's deviational exponent, and $t_{i}$ is the point of inflection on the *x*-axis, which indicates when the timing of a downturn or upturn in the growth rate of the cumulative number of dengue cases occurs. In the fitting result of dengue data, the inflection point can be interpreted as the turning point of dengue infection wave that shows when the West Java infection peak occurs.

The Richards model, shown in Eq. (1), is a widely used growth model which describes many phenomena's temporal growth, such as the cumulative case number of infectious disease [22]. However, unlike the more well-known host-vector compartmental model, the Richards model does not depict the actual process of disease infection. The model has two-fold usefulness in the modeling of infectious disease. First, to estimate the cumulative case number's growth rate, often associated with the outbreak's basic reproduction number; and second, to identify the temporal progression of the infection wave in order to detect the peak (or turning point) of the epidemic [23]. Due to the lack of more detailed human and mosquito data, this study focuses on investigating the outbreak's temporal and spatial progression. A recent study confirms that the Richards model can accurately estimate the epidemic peak with better confidence intervals coverage than three commonly used phenomenological models: exponential, logistic, and delayed logistic [24]. The Richards model is more appropriately applied in data fitting for the reported dengue data with minor errors during observation compared to previous models [25], [26], [27]. Using standard MATLAB (R2017a) software with a nonlinear least squares (NLS) approximation subroutine [28], we obtain two model parameters of dengue epidemiological importance, *r* and $t_{i}$, by fitting the cumulative dengue case number in West Java to the Richards model.

### 2.4 Spatial association analysis

To perform a spatial association analysis, we first determine the overall spatial clusters of dengue cases in each weekly cycle by calculating Moran's Index. The calculation of Moran's Index value [29], [30] is given by:

(2) $$I=\frac{n\sum_{i}\sum_{j}w_{ij}\left(z_{i}-\bar{z}\right)\left(z_{j}-\bar{z}\right)}{\sum_{i}\sum_{j}w_{ij}\sum_{i}{\left(z_{i}-\bar{z}\right)}^{2}}$$

where $w_{ij}=1$, if region *i* and region *j* are adjacent, otherwise, $w_{ij}=0$.

Moran's Index values obtained from Eq. (2) indicate the existence of spatial clusters. The values of *I* usually range from −1 to +1. The index value $\underline{I}=-1/\left(n-1\right)$ is the expectation of *n* randomly distributed spatial units [31]. High values of Moran's Index and significantly above $\underline{I}$ indicate positive spatial autocorrelation and a greater amount of clustering [32]. The index values close to $\underline{I}$ signify probable randomness in the spatial distributions of data. Otherwise, the index values significantly lower than $\underline{I}$ represent negative spatial autocorrelation, and the spatial pattern of data can be more spread than a random pattern.

After determining the possibility of spatial clustering existence of dengue cases, we further used $G^{⁎}$-statistics to examine the clusters at the local level and provide perceptions into the spatial side of the dengue transmission [33], [34]. The calculations of $G^{⁎}$-statistics are presented as follows:

(3) $$G_{i}^{⁎}=\frac{\sum_{j}w_{ij}z_{j}-W_{i}\bar{z}\left(i\right)}{s\left(i\right)\sqrt{\left[n\cdot S_{i}-W_{i}^{2}\right]/\left(n-1\right)}}$$

for all *j*. The symmetric matrix $\left[w_{ij}\right]$ denotes adjacency between point *i* and point *j*. We have $W_{i}=\sum_{j}w_{ij}$ and $S_{i}=\sum_{j}w_{ij}^{2}$ for all *j*. Moreover, $\bar{z}$ and *s* denote mean and variance, respectively.

$$\bar{z}(i)=\frac{\sum_{j}z_{j}}{n-1}\;\;\;s^{2}(i)=\frac{\sum_{j}z_{j}^{2}}{n}-{\left[\bar{z}(i)\right]}^{2}$$

In this study, we calculated the $Z_{G^{⁎}}$ score for each week using the weekly reported dengue cases as the observed variable, started from the beginning of the outbreak in all districts and cities. Using Eq. (3), the value of $Z_{G^{⁎}}$ can be calculated as:

(4) $$Z_{G_{i}^{⁎}}=\frac{G_{i}^{⁎}-E(G_{i}^{⁎})}{\sqrt{V(G_{i}^{⁎})}}$$

where *E* and *V* are given by:

$$E(G_{i}^{⁎})=\frac{W_{i}}{n}\;\;\;V(G_{i}^{⁎})=\frac{W_{i}\left(n-W_{i}\right)}{{\left(n\right)}^{2}(n-1)}\cdot {\left[\frac{s(i)}{\bar{z}(i)}\right]}^{2}$$

The statistic presented in Eq. (4) evaluates the degree of spatial co-patterning of geo-referenced data based on both feature values and feature locations simultaneously from a “local” perspective, allowing the identification of hot spots and cold spots. A high $Z_{G^{⁎}}$ score indicates a hot spot or area with high dengue infection. By contrast, a cold spot refers to a region with a low $Z_{G^{⁎}}$ score, indicating a low number of reported dengue cases.

**Figure 2:**
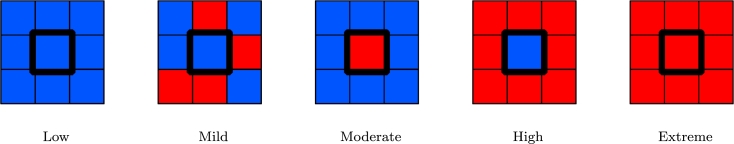
Definition of five spatial risk classifications. Areas with high index scores are shaded in red, whereas areas with low index scores are shown as blue.

Using $Z_{G^{⁎}}$ scores, we classified all districts and cities in West Java into the five epidemiologically distinct risk classifications, from the lowest to the highest, as shown in Fig. 2.

1. **Level 1 (low)**: the target area was considered to be at ‘low risk’ if both the target area and all its surrounding neighbors had low $Z_{G^{⁎}}$ scores.
2. **Level 2 (mild)**: the target area was considered to be at ‘mild risk’ if the target area had low score but some of its neighbors had sporadic high $Z_{G^{⁎}}$ scores.
3. **Level 3 (moderate)**: the target area was considered to be at ‘moderate risk’ and potentially posed some risks to its neighbors when the target area had high score whereas all its surrounding neighbors had low $Z_{G^{⁎}}$ scores.
4. **Level 4 (high)**: the target area was considered to be at ‘high risk’ when either it had low score but all its neighbors had high scores or it had high score and some of its surrounding neighbors also had sporadic high $Z_{G^{⁎}}$ scores.
5. **Level 5 (extreme)**: the target area was considered to be at ‘extreme risk’ when both the target area and all its surrounding neighbors had high $Z_{G^{⁎}}$ scores.

## 3 Results

Among the 49.94 million people living in West Java, 13,169 individuals were reported to be infected by the dengue virus within a select 40 weeks, starting from the first week of October 2019 to the first week of July 2020. Fig. 1a shows that most of the cases (nearly 55%) can be found between the first week of January 2020 and the first week of April 2020. The dengue wave peak, which indicates the highest number of dengue cases, occurred in the fourth week of January 2020 (652 cases) and the second week of March 2020 (660 cases).

Fig. 1b shows the density of reported dengue cases for each city and district in West Java. There are 14 cities/districts (>50%) having a dengue density greater than 25 cases per 100,000 people. The potential dengue exposure regions were distributed and primarily found in the central, northern, and eastern regions. The highest dengue case density was reported in the city of Banjar with 144.57 cases confirmed per 100,000 residents, whereas the district of Sukabumi became the region with the lowest density at 3.12. In contrast, the districts and cities in the western region, including Bodebek and West Priangan, can be considered as the safe zone with a density of less than 25 cases per 100,000 population.

### 3.1 Temporal trend of dengue cases

**Figure 3:**
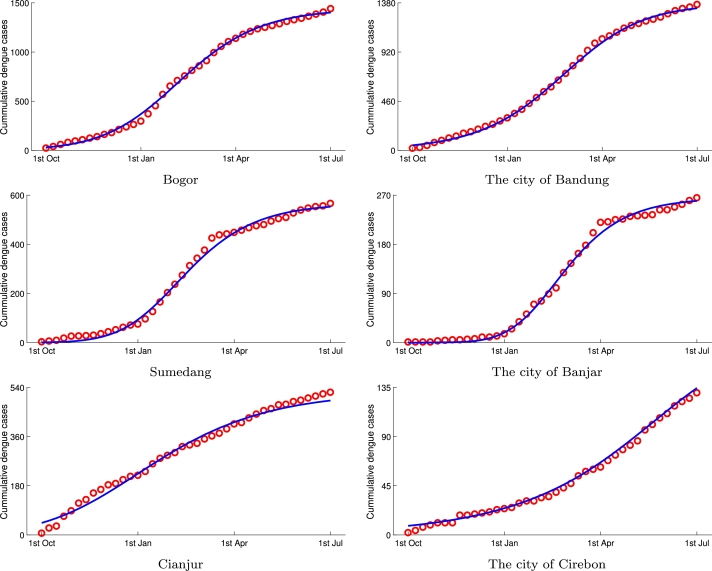
Fitting result of weekly reported dengue data (*red circle*) to Richards model (*blue line*) in some districts of West Java. The district of Bogor and the city of Bandung have the highest case numbers of dengue. The district of Sumedang and the city of Banjar have the highest growth rate parameter. The district of Cianjur has the earliest turning point and the city of Cirebon has the latest turning point.

The weekly data of total confirmed dengue cases for each district and city in West Java were fitted to the Richards model to acquire the model parameters. As can be seen in Fig. 3, the output of the Richards model was well fitted with the dengue data. Table 1 shows the estimated value for each parameter with a 95% confidence interval obtained by 200 bootstrap realization. During these 40 weeks, a total of 13,530 dengue cases were reported, with more than 1,000 cases occurring in the district of Bogor, the district of Cirebon, and the city of Bandung. The growth rate parameter, *r*, ranges from 0.0838 in the city of Cirebon up to 0.6774 in the city of Banjar, whereas generally, the growth rate of dengue in West Java is 0.2095. The range of turning points indicating the timing of upturn or downturn in the rate of dengue cumulative case increase is from week-4 (last week of October) to week-38 (mid-week of June). The earliest turning point occurs in the district of Cianjur, whereas the city of Cirebon has the latest turning point of dengue cases, close to the end of the observation time.

**Table 1:** Summary for estimated parameters by fitting weekly reported dengue data to the Richards model.

Region		Case number *K*	Growth rate *r* (95% CI)	Deviation *μ* (95% CI)	Turning week

Northwestern: Bodebek					
	Depok city	281	0.4343(0.4329,0.4357)	2.5773(2.5725,2.5821)	14
	Bogor	1,440	0.2368(0.2364,0.2371)	1.3865(1.3855,1.3875)	17
	Bekasi	472	0.2206(0.2209,0.2212)	1.8537(1.8511,1.8563)	21
	Bogor city	116	0.2549(0.2546,0.2553)	0.6780(0.6771,0.6789)	22
	Bekasi city	474	0.1611(0.1610,0.1612)	0.8347(0.8326,0.8368)	31


Northern: Purwasuka					
	Subang	547	0.3493(0.3487,0.3499)	2.7770(2.7747,2.7793)	8
	Purwakarta	668	0.2201(0.2198,0.2204)	0.8026(0.8016,0.8036)	21
	Karawang	288	0.1527(0.1525,0.1529)	1.0906(1.0896,1.0916)	22


Northeastern: Ciayumajakuning					
	Indramayu	349	0.1954(0.1952,0.1956)	1.4059(1.4049,1.4069)	16
	Kuningan	296	0.2224(0.2220,0.2227)	1.3990(1.3978,1.4002)	17
	Majalengka	595	0.1754(0.1752,0.1756)	1.9111(1.9075,1.9147)	24
	Cirebon	1,157	0.1331(0.1329,0.1333)	0.8550(0.8541,0.8559)	26
	Cirebon city	184	0.0838(0.0834,0.0842)	0.5694(0.5522,0.5866)	38


Southeastern: East Priangan					
	Pangandaran	136	0.1646(0.1637,0.1654)	1.1135(1.1108,1.1163)	9
	Banjar city	265	0.6774(0.6749,0.6798)	3.3388(3.3314,3.3462)	15
	Ciamis	890	0.4136(0.4120,0.4153)	2.2184(2.2136,2.2232)	17
	Tasikmalaya city	118	0.1914(0.1907,0.1921)	1.1500(1.1477,1.1523)	20
	Garut	923	0.1630(0.1628,0.1632)	1.1152(1.1143,1.1161)	21
	Tasikmalaya	410	0.1191(0.1189,0.1193)	0.7750(0.7706,0.7794)	27


Central: Central Priangan					
	Sumedang	566	0.4698(0.4681,0.4714)	2.5965(2.5912,2.6019)	14
	Bandung	552	0.3319(0.3312,0.3326)	2.0426(2.0403,2.0450)	15
	West Bandung	690	0.2109(0.2107,0.2111)	0.7754(0.7748,0.7760)	18
	Bandung city	1,370	0.1520(0.1519,0.1521)	0.7924(0.7911,0.7936)	23
	Cimahi city	200	0.1164(0.1163,0.1165)	0.4547(0.4541,0.4554)	23


Southwestern: West Priangan					
	Cianjur	522	0.3048(0.3039,0.3058)	2.8587(2.8531,2.8642)	4
	Sukabumi city	44	0.4032(0.4010,0.4054)	2.1699(2.1635,2.1764)	13
	Sukabumi	77	0.1813(0.1810,0.1817)	0.5068(0.5055,0.5081)	18


**West Java**		13,530	0.2095(0.2092,0.2098)	1.4217(1.4202,1.4231)	18

Based on the obtained parameter shown in Table 1, the growth rate *r* seems to be associated with the turning point parameter $t_{i}$. The Pearson correlation test shows the value of correlation ρ=−0.569 with statistically significant *p*-value <0.05, which indicates a strong downhill relationship between these two parameters. A negative correlation coefficient designates a reverse association which describes the extent to which the two variables move in opposite directions. A low value of the growth rate parameter, *r*, signifies that the increase of dengue incidences needs a longer period. Subsequently, the turning point of the dengue wave may possibly occur later. In contrast, a high value of parameter *r* denotes the peak of the dengue incidence wave that occurs earlier in the region. The city of Cirebon $\left(r=0.0838,t_{i}=38\right)$, the city of Bekasi $\left(r=0.1611,t_{i}=31\right)$, and the district of Tasikmalaya $\left(r=0.1191,t_{i}=27\right)$ are some examples of the region in West Java which have low values of growth rate and high turning point parameters.

The dengue wave's turning point in West Java occurred in week-18 which is the last week of January 2020. Since generally, dengue incidences in tropical countries such as Indonesia increase during the rainy period associated with growth in mosquito population, turning point parameter $t_{i}$ can be considered as the peak of dengue cases or the downturn of cumulative case growth rate. There are 14 districts and 5 cities (70.4%) in West Java, which had their turning points ranging from week-14 (first week of January) to week-24 (second week of March). This turning point range can be associated with the wettest period in West Java in which average annual rainfall mainly occurs.

**Figure 4:**
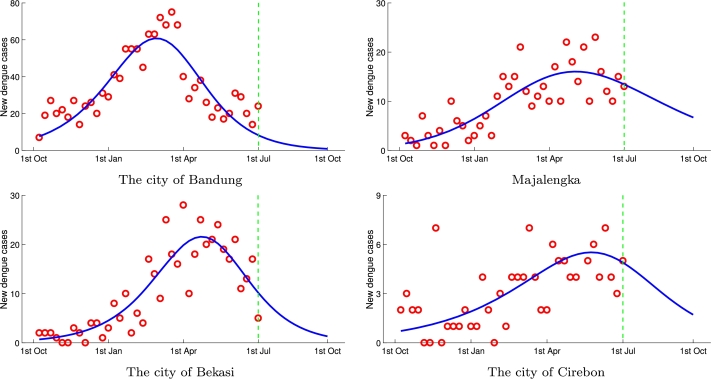
Forecasting 12-week ahead of dengue fever new cases in some region in West Java.

The late turning point means dengue cases are still able to occur in the next few weeks, whereas the early turning point indicates the low number of dengue cases at the end of the cycle of a one year period. Using the set of estimated parameters, predictions for some districts are performed to generate an ensemble of the epidemic curve. We simulated the 12-weeks Richards model ahead of forecasts of new weekly cases to obtain a complete curve in one cycle. Fig. 4 shows the prediction 12-week ahead of dengue fever new cases in some region in West Java. The estimated final epidemic size in West Java is K=13,530, whereas the current reported cases during the 40-week period are 13,169. It means that more than 350 new cases may possibly occur in the next 12 weeks from July to October 2020. Although weekly dengue data was only available until the first week of July 2020, we used the monthly data recorded by the West Java Health Office from July to September (equivalent to 12 weeks) for validation. The total number of dengue cases in West Java reported in these three months was 514, slightly above the predicted result. We also compared the result of prediction and the actual data for the same period in the four regions shown in Fig. 4: the city of Bandung, the district of Majalengka, the city of Bekasi, and the city of Cirebon. Three of these regions showed decent prediction results. West Java Health Office reported 180 new cases in Majalengka, 36 new cases in the city of Cirebon, and 76 new cases in the city of Bekasi. At the same time, the predictions in these regions yielded 194, 51, and 96 dengue cases, respectively. The city of Bandung showed a more inaccurate prediction, where the Richards model forecasted 124 new cases and the real data was 209.

### 3.2 Spatial clustering of dengue cases

**Figure 5:**
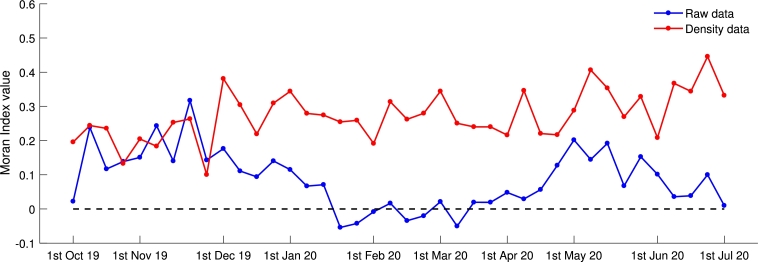
Moran's Index value of weekly raw data and density data of dengue cases in West Java.

In Fig. 5, Moran's Index values, while signifying the presence of spatial clustering, varied considerably during the observation weeks studied. In general, a positively significant Moran's Index coefficient of density data was observed during week 1 until week 40, and its values were higher than the Moran's Index coefficient of raw dengue data. It can be considered that density data has a lower degree of randomness and shows the existence of clusters spatially of dengue cases in West Java. We investigated spatial clustering at a local level using density data of dengue cases for further spatial analysis. The value of *I* fluctuated throughout the observation time, with the highest index evaluated in week-39 (I=0.4458) and the lowest index evaluated in week-9 (I=0.1003).

**Figure 6:**
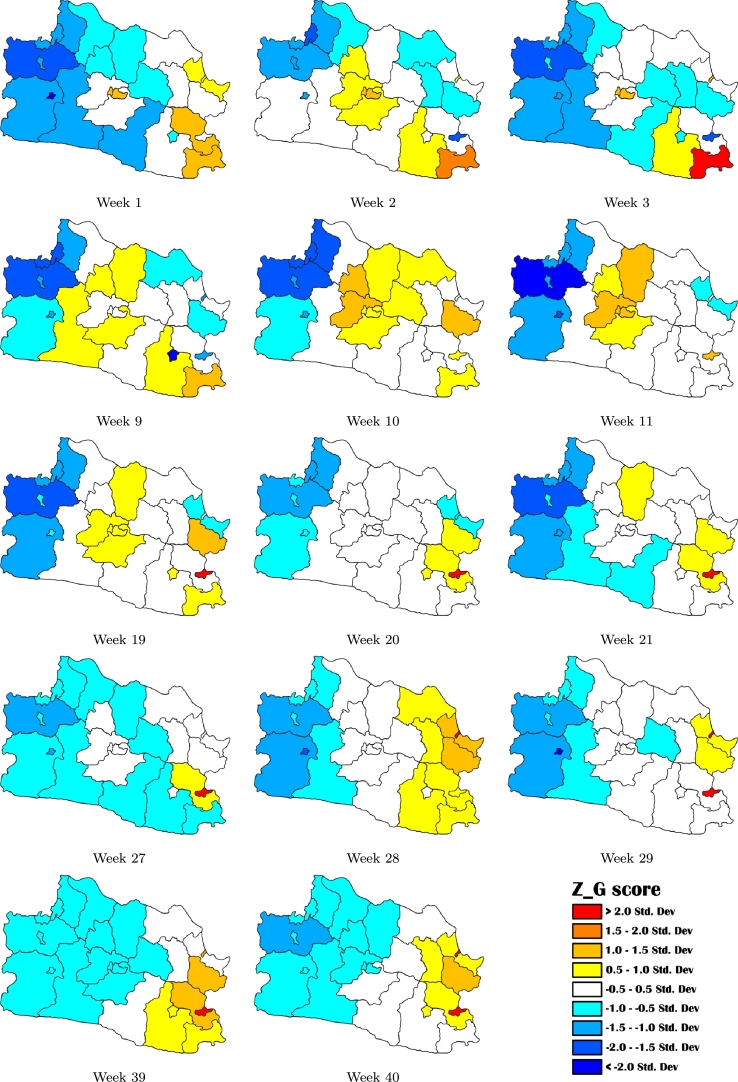
Hot spot analysis of dengue incidences in West Java. Hot spots are shown as yellow-to-red color ($Z_{G^{⁎}}>0.5$ Std.Dev) and cold spots are shown as white-to-blue color ($Z_{G^{⁎}}<0.5$ Std.Dev). Dengue cases evaluated by *G*^⁎^-statistics are denoted for each weekly cycle as a function of dengue incidence rates.

During the study period, the $G^{⁎}$-statistics analysis revealed hot spots distributed across five regions except for the northwestern region of West Java. Fig. 6 depicts the spatiotemporal turnover of high-risk areas of dengue incidence. As can be seen, dengue was notably prevalent in northern (Purwakarta and Subang), southeastern (city of Banjar, Ciamis, Pangandaran and Tasikmalaya) and central (city of Bandung, city of Cimahi, Bandung, West Bandung and Sumedang) regions during the first 20 weeks of the study period. In the next 20 weeks, the southeastern regions remained as hot spots, and all of the regions in northeastern regions, including the city of Cirebon, Cirebon, Kuningan, Majalengka, and Indramayu, were identified as new hot spots. The central and northern regions became cold spots, albeit previously being identified as hot.

Hornsby [35] identified four distinct patterns of spatial diffusion. The growth of spatial phenomenon, which simultaneously widens in every direction, is called expansion diffusion. The direct connection with the adjacent region, not necessarily in all directions at once, is required in the pattern of contagious diffusion. A relocation diffusion describes the dispersing process by shifting to a new region where a new dispersing pattern is possibly different. The diffusion pattern of the phenomenon through a hierarchy of locations, such as urban hierarchy, is called hierarchical diffusion. Furthermore, any or all of these patterns may simultaneously occur. The existence of distinct diffusion patterns of dengue incidences is indicated by the dynamic spread of hot spots among weekly cycles from our mapping. The pattern in the central region during week 1-2 cycle and the southeastern region during week 27-28 indicates correspondence to expansion diffusion. The pattern in the southeastern region on week 1-3 and central region on week 9-11 can be identified as contagious diffusion. In contrast, the cycle during week 9-10 shows very close properties to relocation diffusion in the northeastern region (Kuningan district). The cycle during weeks 39-40, on the other hand, shows diffusion from the southeastern region toward the northeast region.

Table 2 shows the average number of districts or cities for each level in risk classification during ten weeks. Hot spots defined in Fig. 2 are regions with high $Z_{G^{⁎}}$ scores (>0.5 Std.Dev) and are presented in Fig. 6 as areas with yellow-to-red color. Cold spots refer to the regions with low $Z_{G^{⁎}}$ scores (<0.5 Std.Dev) that are shown in Fig. 6 as areas shaded in white-to-blue. All regions are classified according to their own status as a hot spot/cold spot and the status of all the surrounding neighbors. The northwestern and southwestern regions can be considered as safe zones of dengue infection because all of its districts and cities were classified on a low-mild risk level during the 40-week study period. At least one district in the northern region included a high level in the first 20 weeks, but this region showed a low-mild level in the next 20 weeks. The number of high-risk districts or cities in the northeastern region appeared to increase in the last 20 weeks, with 4 districts/cities identified as high-extreme levels in week-31 until week-40. The southeastern region of West Java was the most prevalent region since at least one of its districts/cities was classified in high-extreme level throughout observation time. The high-risk districts or cities in central regions were predominantly found during week-1 until week-20, while surprisingly, they became low-mild during week-31 until week-40.

**Table 2:** The average of the number of regions for each level of risk classification during ten-week periods.

Region	Period	Level 1	Level 2	Level 3	Level 4	Level 5

Northwestern: Bodebek 2 districts, 3 cities	Week 01 - Week 10	4.7	0.3	0.0	0.0	0.0
	Week 11 - Week 20	5.0	0.0	0.0	0.0	0.0
	Week 21 - Week 30	5.0	0.0	0.0	0.0	0.0
	Week 31 - Week 40	5.0	0.0	0.0	0.0	0.0


Northern: Purwasuka 3 districts	Week 01 - Week 10	0.7	1.2	0.0	1.1	0.0
	Week 11 - Week 20	1.0	0.9	0.0	1.1	0.0
	Week 21 - Week 30	2.5	0.4	0.1	0.0	0.0
	Week 31 - Week 40	2.4	0.6	0.0	0.0	0.0


Northeastern: Ciayumajakuning 4 districts, 1 city	Week 01 - Week 10	2.1	1.9	0.5	0.3	0.2
	Week 11 - Week 20	2.7	2.0	0.2	0.1	0.0
	Week 21 - Week 30	1.9	1.7	0.0	0.7	0.7
	Week 31 - Week 40	0.1	0.7	0.1	2.5	1.6


Southeastern: East Priangan 4 districts, 2 cities	Week 01 - Week 10	1.2	2.3	0.4	1.8	0.3
	Week 11 - Week 20	1.7	2.2	0.8	0.9	0.4
	Week 21 - Week 30	1.1	2.2	0.1	1.5	1.1
	Week 31 - Week 40	1.2	1.8	0.3	1.9	0.8


Central: Central Priangan 3 districts, 2 cities	Week 01 - Week 10	0.2	2.7	0.1	1.6	0.4
	Week 11 - Week 20	1.5	1.1	0.0	1.4	1.0
	Week 21 - Week 30	4.6	0.4	0.0	0.0	0.0
	Week 31 - Week 40	3.9	1.1	0.0	0.0	0.0


Southwestern: West Priangan 2 districts, 1 city	Week 01 - Week 10	2.1	0.8	0.0	0.1	0.0
	Week 11 - Week 20	2.3	0.7	0.0	0.0	0.0
	Week 21 - Week 30	3.0	0.0	0.0	0.0	0.0
	Week 31 - Week 40	2.8	0.2	0.0	0.0	0.0

## 4 Discussion

Dengue fever is one of the common acute and notable infectious diseases in West Java. Dengue cases are reported each year and widely distributed in all districts and cities. This research study examined the recent record of dengue virus infection and specifically analyzed the temporal trends of cumulative dengue cases for each region. Furthermore, we investigated the spatial pattern of dengue incidences and identified the potential areas of high infection across the West Java throughout 40-week period.

The trend of confirmed dengue cases significantly increased in West Java from January to March. More than two-thirds of West Java regions had their dengue wave turning point, indicating peaking dengue cases, ranging from the first week of January to the second week of March. This trend clearly coincided with the peak of precipitation level during the rainy season. This result is consistent with other tropical countries' findings, which reported that most dengue infections occurred during the rainy period. Many researchers confirmed that dengue cases were higher during the rainy period compared to the drought period, signifying that precipitation has a significant role in the transmission of the dengue virus. In Southeast Asian regions, Cheong et al. [36] and Sumi et al. [37] indicated that dengue fever cases during a week were related to the precipitation over the previous 26–28 days in Malaysia and 6–7 weeks in the Philippines. Shil [38] identified that northern states in India experience more dengue cases due to high annual rainfall, and Morales et al. [39] confirmed the strong association between rainfall and increase of dengue cases two months later in another South Asian country, Bangladesh. Further, Simard et al. [40] reported that precipitation could maximize the occurrence of larval development sites in both natural and artificial containers in Cameroon, Central Africa. In American tropical countries, a 10-millimeter increase in precipitation was related to an increase in dengue incidence of 6.0% in Brazil [41], 4.1% in Curacao [42], and 2.1% in Mexico [43] in the following month. However, some studies showed no such relationship between dengue incidence and precipitation in other regions. Goto et al. [44] reported that the rainfall does not significantly influence dengue cases in Sri Lanka, while Thammapalo et al. [45] showed a similar result in some provinces in Thailand.

The influence of precipitation on dengue fever risk is confounded by the interactions with human behavior (e.g., water storage) and social-ecological conditions (e.g., housing condition) affecting mosquito abundance. It is generally understood that precipitation is one of the climate factors that strongly influences vector ecology and subsequent dengue infection. The increase of precipitation creates and maintains an important breeding site of the mosquito life cycle for the aquatic stages [46]. Not surprisingly, the abundance of adult mosquito populations and increased mosquito egg collections coincide with the rainy period. Water-holding containers inside and around homes, common in urban and semi-urban environments, are the most critical pupae habitat to complete their development and produce adult mosquitoes [47]. Female mosquitoes lay their eggs on the side of water containers provided by nature (e.g., tree holes and bamboo tubes) or human-made containers (e.g., beverages bottles and water tanks), and the eggs will hatch into larvae after rain. Indeed, *Aedes* eggs can withstand dry conditions for several months on the inner part of the container walls and hatch immediately after being submerged in water [48]. The larvae evolve into pupae and then transform into adults within a few days under favorable environmental conditions. Furthermore, precipitation also influences dengue vector distributions. The mosquito ranges expand during the rainy season (generally wetter) and decrease during the drought period (generally drier). However, a high intensity of rainfall may result in flooding, which flushes outbreeding sites and eradicates the larvae, thus reducing the mosquito population, which could limit the risk of dengue spread.

Through multiple mechanisms influenced by natural and human factors, dengue fever can transmit and spread from the origin of an outbreak into and outside of the areas limited by the flight range of dengue vectors. The spatial distribution analysis enables the visualization of regions with high reported dengue cases and the geographical pattern exploration over the study period. This study showed the existence of dengue case spatial clustering based on Moran's Index value of density dengue data. Furthermore, we identified that northern, central, and southeastern regions of West Java were the high-risk areas during the first 20 weeks of observation time. In contrast, the high-risk areas were pinpointed in southeastern and northeastern regions throughout the next 20 weeks.

In our study, the high-risk dengue infection areas were distributed in areas with low-to-high population densities but mostly living in a densely populated area. Pangandaran and Ciamis in the southeast as well as Kuningan in the northeast were examples of districts with low resident densities, less than 1,000 per square kilometers, that identified as hot spots with high reported dengue infection cases. This finding corroborates the study in Thailand, which found that dengue fever is more prevalent in rural than in urban and semi-urban areas [49]. Furthermore, this study also found that most of the regions in the central, northeastern, and northern areas of West Java, considered as hot spots, were districts/cities with dense human populations. Bandung city (14,970 p/km^2^), Cimahi city (15,798 p/km^2^) and Bandung (2,167 p/km^2^) in the central region, Cirebon city (8,627 p/km^2^) and Cirebon (2,244 p/km^2^) in the northeastern region, and Purwakarta (1,177 p/km^2^) in the northern region reported a high number of dengue incidences.

Numerous studies found a strong correlation between the high-risk areas of dengue infection and densely urbanized areas. Environmental and social features of these areas are likewise seen to affect the number of dengue incidences. High population densities enable rapid urbanization and intensive human mobility that might support virus distribution and increase the possibility of contact between dengue vectors and humans [50]. Due to the lack of decent water infrastructure, congested human settlements can offer favorable breeding opportunities for mosquitoes using a wide range of natural and artificial water-filled containers for egg-laying [51]. Environmental degradation, urban land expansion, and poor sanitary conditions facilitate the favorable conditions of the parasite infection, which may assist in a higher risk of dengue outbreaks in this region. Besides, water-storing habits for domestic use inside the house due to the lack of water supply, especially throughout the drought period, escalate the chance of mosquito oviposition and house-to-house mosquito dispersion, which causes the excess of the dengue viruses [52]. However, the cold spots of dengue infection were identified in the northwestern region, including Bekasi, Bogor, and Depok, notwithstanding this being one of the densely populated areas in West Java. A similar finding was reported by Sirisena et al. [53] in Sri Lanka that dengue vector distribution seems to be limited by the high altitude. Unlike in Sri Lanka, the specific factors underlying this condition in West Java are unclear, but it looks to be influenced by dengue prevention and control by respective local health authorities.

This study has some limitations. Most large-scale observational studies have general methodological issues, e.g., confounding, bias, and inaccuracy. Confounding factors at the individual-level (e.g., behavior, nutrition status, immunity), household-level (e.g., vector control, water storage habits, socioeconomic), or environmental condition for each district and city were not included in this research. One source of bias may be due to unreported cases since we used passive surveillance dengue data, which may possibly underestimate the actual dengue cases in some regions, especially in rural regions. Further, inaccuracy may appear as the consequence of the lack of detailed dengue data. In addition, the other limitation is that the Richards model in this study was only used for one cycle (year), so it has predictive capabilities limited in that cycle and is unable to be applied for the prediction of dengue cases in the next cycle (year).

## 5 Conclusion

In summary, this paper demonstrates the temporal trend and spatial clustering of dengue cases in West Java. Dengue infection is commonly reported in this region, and the trend of dengue cases is likely to escalate significantly during the rainy season. The high-risk areas are detected in not only urban and semi-urban areas distributed in the central, northeastern, and northern regions but also rural areas in the southeastern region. These findings might be due to recent changes of social, ecological, and demographic factors, for example, human behavioral change, movement of population, water tank installation, as well as environmental changes such as climate change and urbanization in West Java. Socio-demographic and ecological factors provide natural habitats for mosquitoes, and increased travel and transport increase the chance of dengue virus importation. Therefore, all health professionals need to be aware of dengue risk, particularly in dengue endemic areas, to minimize the dengue burden and protect the population. It is necessary to implement mosquito control measures in the high-risk areas to prevent the vector abundance. Local health offices should take early preventive measures, conduct enhanced surveillance, and prioritize resource allocation in the high-risk areas to reduce the risk of epidemics. Additional implications of the study include future investigations in identifying risk factors and effective interventions in the high-risk areas for dengue management.

## Declarations

### Author contribution statement

Ilham Saiful Fauzi: Conceived and designed the experiments; Performed the experiments; Analyzed and interpreted the data; Contributed reagents, materials, analysis tools or data; Wrote the paper.

Nuning Nuraini: Conceived and designed the experiments; Analyzed and interpreted the data; Contributed reagents, materials, analysis tools or data; Wrote the paper.

Regina Wahyudyah Sonata Ayu: Performed the experiments; Analyzed and interpreted the data; Wrote the paper.

Bony Wiem Lestari: Analyzed and interpreted the data; Contributed reagents, materials, analysis tools or data; Wrote the paper.

### Funding statement

Nuning Nuraini was supported by 10.13039/501100015689Institut Teknologi Bandung (PPMI Research Grant ITB 2021 No. 62/IT1.C02/SK-TA/2021).

### Data availability statement

The authors do not have permission to share data.

### Declaration of interests statement

The authors declare no conflict of interest.

### Additional information

No additional information is available for this paper.
